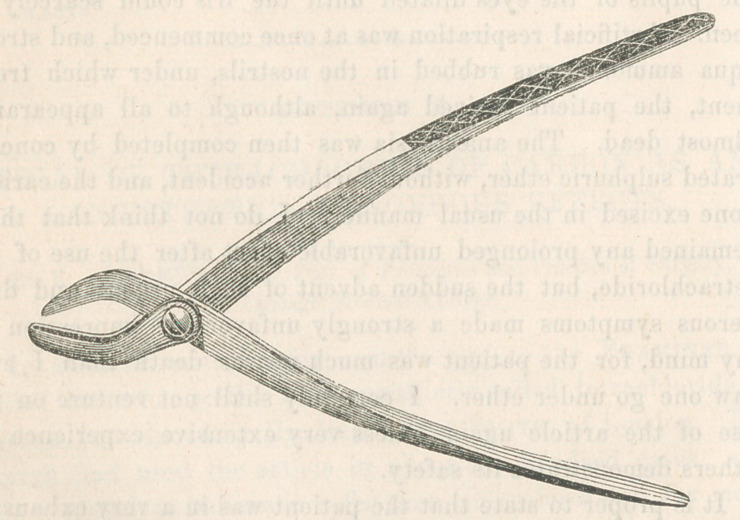# A New Ecraseur Forceps

**Published:** 1867-12

**Authors:** E. Andrews

**Affiliations:** Prof. of Principles and Practice of Surgery, Chicago Medical College


ARTICLE LI.

### A NEW ÆCRASEUR FORCEPS.

By E. ANDREWS, M.D., Prof. of Principles and Practice of Surgery, Chicago Medical College.

Nearly a year ago, I devised an instrument, intended as a substitute for the ecraseur, which I named the ecraseur forceps. I afterwards saw an instrument, invented by Dr. SMITH, partly involving the same principle. The form which I had adopted was quite different from the other, and offerred several points of superiority over it. The subjoined wood-cut gives a correct idea of its construction.

It consists of a strong pair of forceps, of which the length of the jaws is to that of the handles as one to four. One jaw is thick, and has a slot through nearly its entire length; the other is thinner, and plays through the slot, as the chain of the ecraseur plays through the opening in that instrument. The jaws are bent towards each other, in such a way that the beaks pass first, enclosing the tissue to be crushed in a diamond-shaped loop, which grows smaller in all directions as the jaws are pressed together. They act, therefore, very much like the chain of the ecraseur, in gathering all the tissue towards a common centre, under a powerful compression, before they crush it asunder. The edges of the jaws are rounded, to prevent them from cutting like scissors.

This instrument has many advantages over the chain ecraseur, among which is, the ease of its application to almost all sorts of cases. For instance, in removing tumors from the side of the tongue. The chain of the ecraseur has to be drawn through the tongue by a needle and thread, from which the chain must then be detached and attached to the instrument, before the operator can proceed; but in the ecraseur forceps no such trouble is necessary. The surgeon simply applies his instrument to the tongue, like a pair of scissors; the beaks sink through the organ, at the exact spot where they are placed; and the enclosed tissue is instantly under the gathering compression of the jaws. A second application, on the other side of the tumor, completes its removal. In removing piles, it operates admirably well. In case of a large tumor, whose pedicle cannot be wholly grasped at once by the jaws, it is simply necessary to take it at two or three bites. A collateral advantage of these instruments is their cheapness. They are made by Tolle & Degenhardt, Chicago, for from three to four dollars, while a chain ecraseur costs from ten to twenty dollars, according to size. The only objection to it is this:—Ecraseur work should always be done slowly. It is by the *gradual* compression that one makes sure of closing the vessels so as to avoid hemorrhage. The chain ecraseur tightens with a screw, whose turning requires time, and thus compels deliberation; while in the forceps form, it is in the power of a careless surgeon to grip the handles quickly together, and make the sundering of the tissues too sudden. In using it, the surgeon should compress the handles slowly and gradually, allowing about a minute to each act of crushing.

## Figures and Tables

**Figure f1:**